# Fringe Projection Profilometry Based on Saturated Fringe Restoration in High Dynamic Range Scenes

**DOI:** 10.3390/s23063133

**Published:** 2023-03-15

**Authors:** Hongru Li, Hao Wei, Jiangtao Liu, Guoliang Deng, Shouhuan Zhou, Wenwu Wang, Liang He, Peng Tian

**Affiliations:** 1 College of Electronics and Information Engineering, Sichuan University, Chengdu 610065, China; 2 School of Mechanical Engineering, Sichuan University, Chengdu 610065, China

**Keywords:** fringe projection profilometry, three-dimensional measurement, high dynamic range, fringe saturation, saturated fringe restoration

## Abstract

In high dynamic scenes, fringe projection profilometry (FPP) may encounter fringe saturation, and the phase calculated will also be affected to produce errors. This paper proposes a saturated fringe restoration method to solve this problem, taking the four-step phase shift as an example. Firstly, according to the saturation of the fringe group, the concepts of reliable area, shallow saturated area, and deep saturated area are proposed. Then, the parameter *A* related to the reflectivity of the object in the reliable area is calculated to interpolate *A* in the shallow and deep saturated areas. The theoretically shallow and deep saturated areas are not known in actual experiments. However, morphological operations can be used to dilate and erode reliable areas to produce cubic spline interpolation areas (CSI) and biharmonic spline interpolation (BSI) areas, which roughly correspond to shallow and deep saturated areas. After *A* is restored, it can be used as a known quantity to restore the saturated fringe using the unsaturated fringe in the same position, the remaining unrecoverable part of the fringe can be completed using CSI, and then the same part of the symmetrical fringe can be further restored. To further reduce the influence of nonlinear error, the Hilbert transform is also used in the phase calculation process of the actual experiment. The simulation and experimental results validate that the proposed method can still obtain correct results without adding additional equipment or increasing projection number, which proves the feasibility and robustness of the method.

## 1. Introduction

Fringe projection profilometry (FPP) [1] is an important three-dimensional (3D) measurement technique with the advantages of noncontact, high accuracy, high speed, and simple instrumentation; it is widely used in industrial modeling, medical imaging, heritage conservation, etc. The basic principle is to project periodically changing sinusoidal fringe patterns onto the measured object, and the object’s height will modulate the fringes and cause distortion. Then, a camera is utilized to capture the distorted fringe patterns, and the phase of the object, which represents its height, is obtained using phase retrieval methods [2,3,4,5]. Generally, FPP is very effective for Lambertian objects. However, due to the difference in reflectivity of different parts of ordinary objects and the camera’s limited dynamic range, the captured fringes may suffer underexposure or overexposure, thus losing the sinusoidal characteristics of the fringe. Very dark fringes are easily affected by noise, and very bright fringes tend to reach the saturation threshold, which may result in large errors in both cases. Therefore, determining how to carry out 3D measurement in high dynamic range (HDR) scenes has become an important research topic. Many effective methods have been proposed to improve the dynamic range of FPP. Current high dynamic 3D measurement techniques can be classified into two categories [6]: device-based techniques and algorithm-based techniques.

The device-based techniques mainly involve adjusting the instrumentation in the optical path, such as cameras or projectors, to reduce the light intensity entering the camera. Proll et al. [7] used color cameras with different sensitivities to color in the R, G, and B channels to obtain images with different dynamic ranges. Still, this method has a slight improvement in dynamic range. Ri et al. [8] applied a digital micromirror device (DMD) camera to improve the system’s dynamic range, and this method requires high equipment requirements and is difficult to adjust. The multiexposure method [9] does not require such complex equipment adjustments. Its basic principle is to project multiple groups of fringe patterns to the measured object. By adjusting the camera’s exposure time, each group of fringe patterns captured has a different brightness. The fringe group with the maximum value but not saturated is used for phase calculation. This method can avoid saturation of the fringes, and the phase of each position is calculated using the best-exposed fringes, reducing the impact of noise on low-exposure positions. However, due to the need to capture many images, this method only applies to static objects. The basic principle of adjusting the projector to improve the dynamic range [10] is to control the projector to automatically adjust the intensity of the projected light according to the illumination of the fringes to avoid saturation, and this method requires preprojected images to sense the reflectivity of objects, which also takes more time. Adding additional devices [11,12,13], such as polarizers or filters on the optical path, can also eliminate saturation points and improve the fringe contrast ratio, achieving high dynamic range imaging. Although this method avoids saturation, it leads to a low signal-to-noise ratio in the low-illumination region, and the fringes are susceptible to noise interference. Other improved techniques [14,15,16,17] based on the above methods have also been proposed, further improving the effect and robustness.

The algorithm-based techniques do not require equipment adjustments but rather calculate the ideal phase directly using saturated fringes. This is more applicable when equipment changes cannot be made or additional equipment is unavailable. Jiang et al. [18] proposed using inverted fringe patterns to help phase calculation when the other fringe patterns are saturated. Tan et al. [19] analyzed the saturated signal. They designed a phase error correction method combining Fourier analysis and Hilbert transform, in which they low-pass-filtered the saturated fringes in the frequency domain to convert them into smooth sinusoidal fringes. Then, Hilbert transform was used to reduce the error further, but this method leads to the loss of high-frequency details of the object. In recent years, deep learning techniques have been developed rapidly and performed very well in image processing. Researchers have used deep learning approaches to solve the fringes saturation problem and improve the dynamic range of FPP [20,21]. As a data-driven approach, deep learning techniques require a large number of datasets for training, and the accuracy and generalization ability of the network is difficult to balance.

Through the above introduction, it can be noted that the device-based techniques need to introduce additional equipment or more projection numbers. This paper proposes an algorithm-based method to restore saturated fringes, which can effectively improve measurement efficiency and potentially apply to high-speed measurement. The key is to consider a parameter *A* related to the object’s reflectivity and use the local continuity assumption of reflectivity to restore the unknown *A* values with the known *A* values. The concepts of reliable area, shallow saturated area, and deep saturated area are introduced to achieve this goal. The reliable area can be obtained by the saturation of the fringe group, while the shallow and deep saturated areas can be obtained by using morphological operations to the reliable area. *A* can be calculated in the reliable area, that is, as a known quantity. It can be used to interpolate the shallow and deep saturated areas using cubic spline interpolation (CSI) [22] and biharmonic spline interpolation (BSI) [23], respectively, to restore the entire *A*. Then, based on the relationship between the fringes, the saturated fringe is restored by using the unsaturated fringe, and CSI completes the parts that still cannot be restored in this process. To further reduce the influence of nonlinearity in practical experiments, the Hilbert transform can be applied to offset the error. Simulation and experimental results validate the feasibility and robustness of the proposed method.

## 2. Principle

After projecting sinusoidal fringe patterns to the object, the images captured from a camera can be modeled as [9]

(1) $$I\left(x,y\right)=\alpha \left\{r\left(x,y\right)\left[a\left(x,y\right)+b\left(x,y\right)\cos\varphi \left(x,y\right)+a_{1}\left(x,y\right)\right]+a_{2}\left(x,y\right)\right\},$$

where α is the sensitivity of the camera, $\left(x,y\right)$ is the pixel coordinate of the camera, $r\left(x,y\right)$ is the reflectance of the object, $a\left(x,y\right)$ is the average intensity, $b\left(x,y\right)$ is the intensity modulation, $\varphi \left(x,y\right)$ is the phase related to the object’s height, $a_{1}\left(x,y\right)$ is the ambient light shining on the object, and $a_{2}\left(x,y\right)$ is the ambient light that enters the camera directly. To ensure higher quality fringes, the ambient light $a_{2}\left(x,y\right),$ is required to be dark enough that it can be ignored, then we further simplify Equation (1):

(2) $$\begin{array}{l} I\left(x,y\right)=A+B\cos\varphi \left(x,y\right)\\ A=\alpha r\left(x,y\right)\left[a\left(x,y\right)+a_{1}\left(x,y\right)\right]\\ B=\alpha r\left(x,y\right)b\left(x,y\right) \end{array}.$$

Currently, 8-bit cameras are commonly used, and their pixel values are less than or equal to 255 ($=2^{8}-1$). Therefore, this paper will take an 8-bit camera as an example for further discussion. Other cameras with different bit numbers are similar. In high dynamic scenes, noise often affects the underexposed area, which will bring greater phase fluctuations. One strategy is increasing the exposure time of the camera to improve the brightness of the underexposed area, which will saturate the normally exposed area. The saturation signal can be expressed as

(3) $$I^{t}\left(x,y\right)=\left\{\begin{array}{c} \begin{array}{cc} I\left(x,y\right) & I\left(x,y\right)<255 \end{array}\\ \begin{array}{cc} 255 & I(x,y)\geq 255 \end{array} \end{array}\right..$$

**Figure 1:**
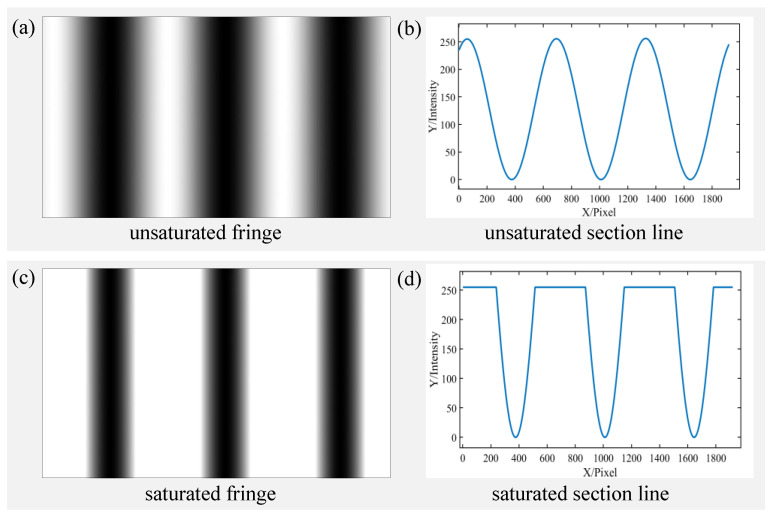
Examples of unsaturated and saturated fringes. Panels (**a**,**b**) are unsaturated fringe and its section line, respectively. Panels (**c**,**d**) are saturated fringe and its section line, respectively.

As shown in Figure 1, the first row is an unsaturated fringe, and the sinusoidal trend of its section line is normal. The second row is a saturated fringe, and the part exceeding the camera response 255 is truncated, whose section line produces a flat top.

In *N*-step phase-shifting profilometry (PSP), the captured deformed fringe patterns can be represented as

(4) $$I_{n}\left(x,y\right)=A+B\cos\left[\varphi \left(x,y\right)+\frac{2\pi n}{N}\right],$$

where n=1,…,N is the phase-shifting index. This paper uses the four-step PSP as an example to introduce the method. That is, when N=4, each fringe pattern can be expressed as

(5) $$\begin{array}{l} I_{1}=A-B\sin\varphi \\ I_{2}=A-B\cos\varphi \\ I_{3}=A+B\sin\varphi \\ I_{4}=A+B\cos\varphi \end{array}.$$

It is easy to find that in those four patterns, $I_{1}$ and $I_{3}$ are symmetric to *A*, and, similarly, $I_{2}$ and $I_{4}$ are also symmetric to *A*. Based on this finding, when *A* is known, if a fringe is saturated at a certain position, its symmetrical fringe is not saturated. We can attempt to use the unsaturated fringe to restore the saturated fringe. Thus, the restored fringes in the saturated area Ω can be expressed as

(6) $$\begin{array}{l} I_{1\Omega }^{r}=2A-I_{3}\\ I_{2\Omega }^{r}=2A-I_{4}\\ I_{3\Omega }^{r}=2A-I_{1}\\ I_{4\Omega }^{r}=2A-I_{2} \end{array}.$$

**Figure 2:**
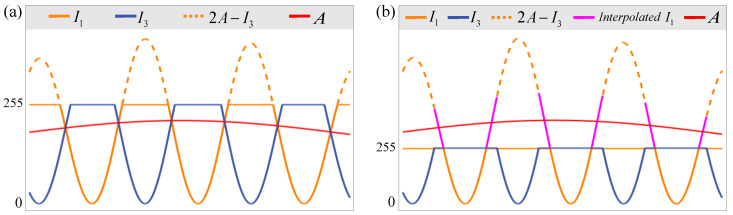
Two saturation fringe restoration strategies with different saturation. Assume that *A* is known in both scenarios. (**a**) *A* < 225, all saturated areas of *I*_1_ can be restored through unsaturated areas of *I*_3_. (**b**) *A* ≥ 225, only a part of saturated areas of *I*_1_ can be restored through unsaturated areas of *I*_3_, and the rest can be further obtained by interpolation.

To illustrate this idea more clearly, as shown in Figure 2, only $I_{1}$ and $I_{3}$ are selected, for example. According to the degree of saturation, and assuming that *A* is known, it can be divided into small-saturation (A<255) and large-saturation (A≥255) scenarios, as shown in Figure 2a,b, respectively. In Figure 2a, it can be observed that the values exceeding 255 in both $I_{1}$ and $I_{3}$ are truncated. However, $I_{3}$ is not saturated in the area where $I_{1}$ is saturated, so $I_{3}$ can be used to restore $I_{1}$ by Equation (6), as shown by the orange dotted line. It can also be noted in Figure 2b that $I_{1}$ and $I_{3}$ are truncated. The difference is that $I_{3}$ can only restore a part of saturated areas of $I_{1}$ due to the large saturation. The remaining part of $I_{1}$ that is not restored can be interpolated, as shown by the solid magenta line. It is worth noting that this part of $I_{1}$ is the same for $I_{3}$, so this part of $I_{3}$ does not need to be interpolated again and can be completed using Equation (6). Therefore, for symmetrical fringes, only one of them needs to be interpolated.

Through the above discussion, it can be noted that *A* is a crucial quantity in this process. If it is possible to obtain *A*, the fringes in the saturated area can be restored. However, in fact, *A* can only be solved in some cases, and its theoretical value cannot be solved in other cases. Table 1 lists all fringe saturation cases in the four-step PSP at the same position. All cases can be divided into five categories:

(1) All *I*_1_, *I*_2_, *I*_3_, *I*_4_ are unsaturated. *A* can be solved by

(7) $$A=\frac{I_{1}+I_{2}+I_{3}+I_{4}}{4}.$$

(2) Only one fringe is saturated, and the other three are unsaturated. According to different fringe saturation combinations, if *I*_1_, *I*_3_ are unsaturated, and one of *I*_2_, *I*_4_ is saturated, according to Equation (5), *A* can be calculated by

(8) $$A=\frac{I_{1}+I_{3}}{2}.$$

Similarly, if *I*_2_, *I*_4_ are unsaturated, and one of *I*_1_, *I*_3_ is saturated, then

(9) $$A=\frac{I_{2}+I_{4}}{2}.$$

(3) Two fringes are saturated, and the other two are unsaturated. Theoretically, there are six combinations, but according to Equation (5), two will not occur. Here is simple proof. If the *I*_1_, *I*_3_ < 225 and *I*_2_, *I*_4_ ≥ 225 can hold, then if (*I*_1_ + *I*_3_)/2 = *A* < 225, and (*I*_2_ + *I*_4_)/2 = *A* ≥ 225, these two results are contradictory, so the assumption is not established. Similarly, the case that *I*_2_, *I*_4_ < 225 and *I*_1_, *I*_3_ ≥ 225 will not appear. The remaining four scenarios are possible. However, in all combinations, *A* cannot be calculated.
(4) Three fringes are saturated, and only one is unsaturated. All four combinations cannot calculate *A*.
(5) All *I*_1_, *I*_2_, *I*_3_, *I*_4_ are saturated. It is obviously impossible to calculate *A*.

From the above analysis, it can be known that only the first two cases can calculate *A*. In other words, even if saturation occurs, only one fringe is saturated, and *A* can be calculated. For the convenience of subsequent discussion, the area of cases 1 and 2 that can accurately obtain *A* is called a reliable area. Obviously, the value range of *A* is less than 255. Although *A* cannot be obtained in cases 3 to 5, it is assumed that the true value of *A* is $A^{\prime }$. When $A^{\prime }<255$, the area is called a shallow saturated area, and the area with $A^{\prime }\geq 255$ is called a deep saturated area.

**Figure 3:**
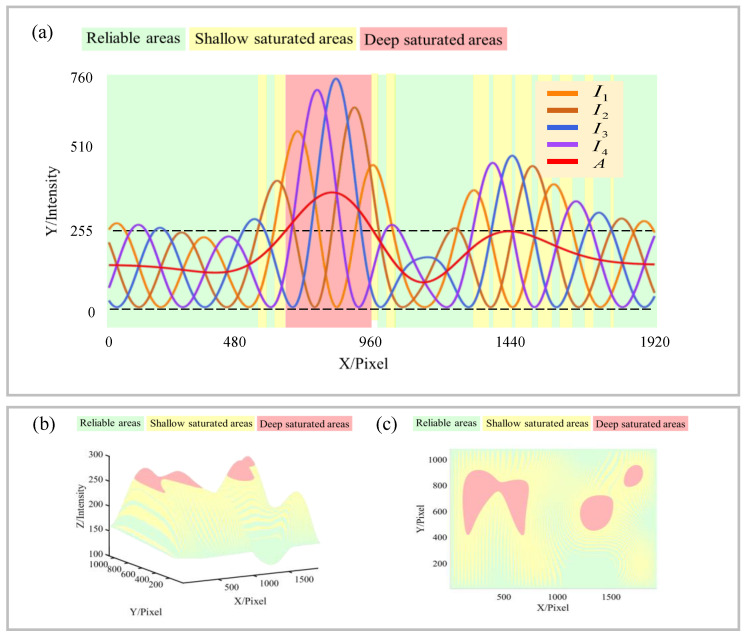
Examples of divisions of reliable area, shallow saturated area, and deep saturated area. (**a**) Example of one-dimensional area division. Panels (**b**,**c**) are examples of two-dimensional area division. Panel (**c**) is the top view of (**b**). All subgraphs use green, yellow, and red to represent reliable areas, shallow saturated areas, and deep saturated areas, respectively.

As shown in Figure 3, the area division examples of reliable area, shallow saturated area, and deep saturated area are shown. The area with green background refers to the reliable area, the yellow refers to the shallow saturated area, and the red refers to the deep saturated area. Figure 3a is an example of a one-dimensional area division, and Figure 3b,c are examples of two-dimensional area division, where Figure 3c is the top view of Figure 3b. It can be noted that when *A* exceeds 255, it is a deep saturated area. When *A* is less than 255, it may be a reliable area or a shallow saturated area, depending on the saturation of the fringe group.

The problem now is determining how to find *A* in shallow and deep saturated areas. It can be seen from Equation (2) that *A* is related to α, r, a, and $a_{1}$. In the actual measurement system, α, a, and $a_{1}$ are generally stationary values, so r has a greater impact on *A*. The reflectivity r mainly depends on the nature of the object itself (surface condition), as well as the wavelength and angle of incident light. When an FPP system is determined, the reflectivity distribution is determined. Although the light intensity sampled by the camera is discrete, the reflectivity distribution can be considered continuous for general physical objects. Therefore, the continuity assumption can be used to interpolate where *A* cannot be calculated.

**Figure 4:**
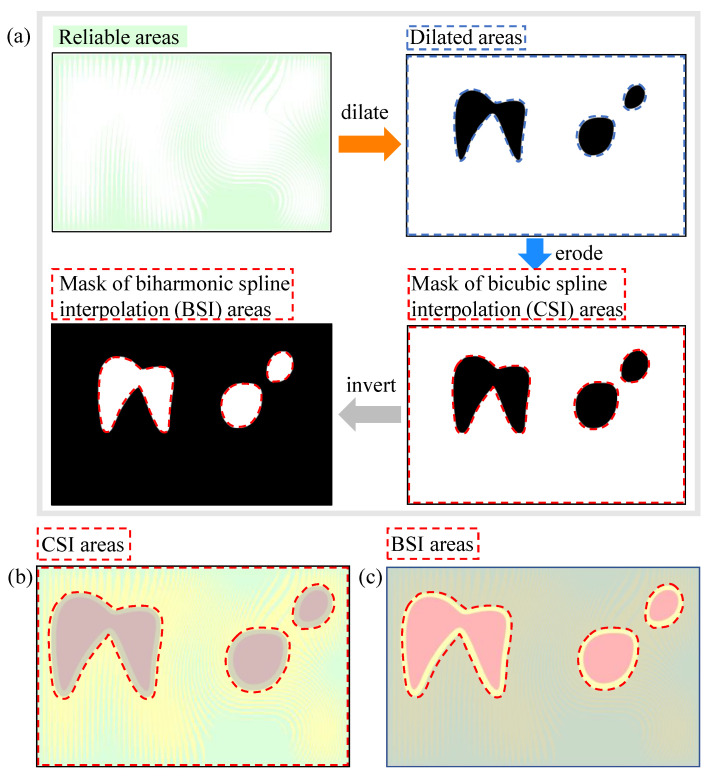
Examples of the formation of cubic spline interpolation (CSI) area and biharmonic spline interpolation (BSI) area. (**a**) Dilation and erosion of the reliable areas to obtain CSI and BSI areas. (**b**) Place the mask of CSI areas on the division map of *A*. The bright areas surrounded by red dotted lines represent CSI areas. (**c**) Place the mask of BSI areas on the division map of *A*. The bright areas surrounded by red dotted lines represent BSI areas.

This paper proposes a strategy to restore *A* using CSI and BSI. It can be noted from Figure 3 that the reliable area and the shallow saturated area are basically interlaced, while the deep saturated area is generally isolated. The *A* values in the reliable area can be used to interpolate the *A* values in the shallow and deep saturation areas. The specific operations are as follows, as shown in Figure 4a:

(1) Obtain reliable areas from fringe patterns.
(2) After determining the reliable area, dilate the mask to obtain the dilated area. The dilation distance should be more than half of the fringe period so that the shallow saturated areas can be connected.
(3) The above dilated area shall be eroded, and the erosion distance shall exceed the dilation distance to exclude the deep saturated area as far as possible (from another perspective, this will leave more reliable points for interpolation in the BSI area). This gives the CSI area.
(4) Invert the CSI area to obtain the BSI area.

Overlay the mask of CSI and BSI areas on Figure 3c to obtain Figure 4b,c, respectively. It can be noted that the CSI area includes most shallow saturated areas and reliable areas, while the BSI area includes all deep saturated areas and a few shallow saturated areas and reliable areas. The CSI area and BSI area both have reliable *A*, so the corresponding interpolation method can be used to obtain the interpolated results.

It can be seen that the above methods use two interpolation methods to restore *A*, so it is necessary to explain the necessity of selecting two interpolation methods. The CSI is a way of finding a curve that connects data points with a degree of three or less, while the BSI is an interpolation of irregularly spaced two-dimensional data points. If only CSI is selected for full field interpolation, the deep saturated area, as shown in Figure 3b, will have a flat top effect because there is no reliable *A* in the inner neighborhood of the deep saturated area, which will lead to interpolation failure. On the other hand, if only BSI is selected for full field interpolation, due to the algorithm characteristics of BSI, it needs to generate the matrix order that depends on the number of reliable points. In the case of a typical camera resolution of 1920 × 1080, the matrix order will be very large, far exceeding the size of personal computer memory. Through the mask selection of the BSI area, the number of points requiring BSI is significantly reduced, thus meeting the operational requirements, and, based on the neighborhood interpolation principle, although the number of points interpolated is reduced, the correct results can still be obtained, which just solves this contradiction. Therefore, the CSI method is preferred for the interlaced reliable area and shallow saturated area, while the BSI method is preferred for the relatively isolated deep saturated area.

Based on the above discussion, it will be possible to obtain *A* for full field, and the specific operation steps are summarized as follows:

(1) Determine the reliable area and obtain the CSI and BSI areas.
(2) In CSI and BSI areas, the corresponding interpolation method is used to obtain the interpolated *A*.
(3) Merge interpolation results of the two areas to restore full *A*.

**Figure 5:**
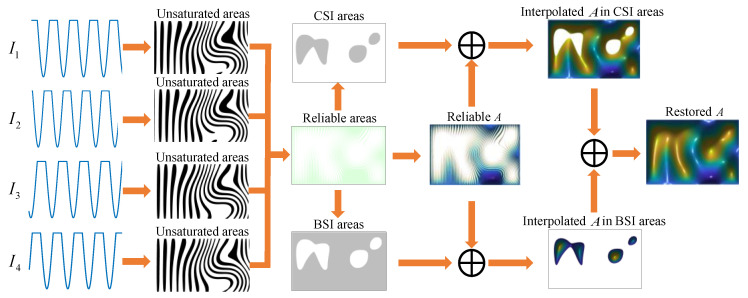
Flowchart of restoration of *A*.

The whole process is shown in Figure 5.

After *A* is restored, the saturated fringes can be restored by Equation (6). As shown in Figure 2, when A<255, the inverted fringe can completely restore the saturated fringe. When A≥255, only the part at the top of the saturated fringe can be restored, and the rest can be interpolated by the CSI method; then, the same part of the symmetrical fringe can be further restored. After all the captured fringes are restored, the phase of the object can be calculated using the phase calculation formula [3].

## 3. Simulation

**Figure 6:**
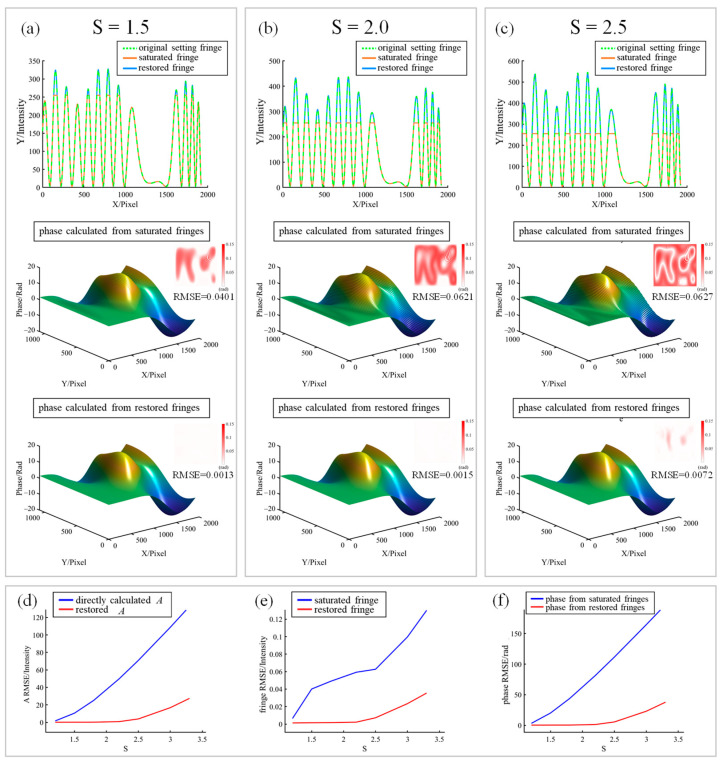
Simulation verification of the proposed method. Panels (**a**–**c**) are the cases when *S* = 1.5, 2.0, 2.5, respectively. The first row is the comparison of the section lines of the original setting fringe, saturated fringe, and restored fringe. The second row is the comparison of the spectra of the original, saturated, and restored cases. The third row is the phase calculated directly from the saturated fringes, and the fourth row is the phase calculated using the restored fringes. The small figures at the upper right corners of the second and third rows indicate the error maps. (**d**) Error comparison between the directly calculated *A* and restored *A* changing with *S*. (**e**) Error comparison between the saturated fringe and restored fringe changing with *S*. (**f**) Error comparison between the phases obtained directly from the saturated fringes and those from restored fringes changing with *A*.

Numerical simulations were performed to verify the feasibility of the proposed method. The saturated fringes are set by $I=S\left[A+B\cos\left(\varphi \right)\right]$, where S is the saturation coefficient. When S<1, the fringe is not saturated. When S≥1, the fringe value will be greater than 255, resulting in saturation. For the part exceeding 255, it will be truncated to 255 according to Equation (3). As shown in Figure 6, the three cases of S=1.5, 2.0, 2.5 are simulated. The first row of Figure 6 is the comparison of the section lines of the original setting fringe, saturated fringe, and restored fringe; it is clear that saturated fringes produce saturation truncation, and the proposed method successfully restores the truncated part. The second row is the comparison of the spectra of the original, saturated, and restored cases, which are obtained by Fourier analysis [19,24]. It can be observed that the higher harmonics in the restored fringes were suppressed. The third row is the phase calculated directly using the saturated fringes, while the fourth row is the phase calculated using the restored fringes. The small figures in their upper right corners are the error maps with root mean square error (RMSE) attached. It can be clearly noticed that with the increase of saturation coefficient, the phase fluctuation calculated by using saturated fringes also gradually increases. However, the phase calculated using the restored fringes mostly stayed the same. Their error maps can also reflect this. To further confirm the robustness of the results obtained by this method under different saturation coefficients, the comparisons of restored *A*, restored fringe, and restored phase with their saturation conditions are studied, as shown in Figure 6d–f, respectively. The comparison results show that the results obtained by this method are much better and more robust than those of the untreated cases from three aspects. There is almost no error when S<2, and the error will slowly increase when *S* exceeds 2.

## 4. Experiment

**Figure 7:**
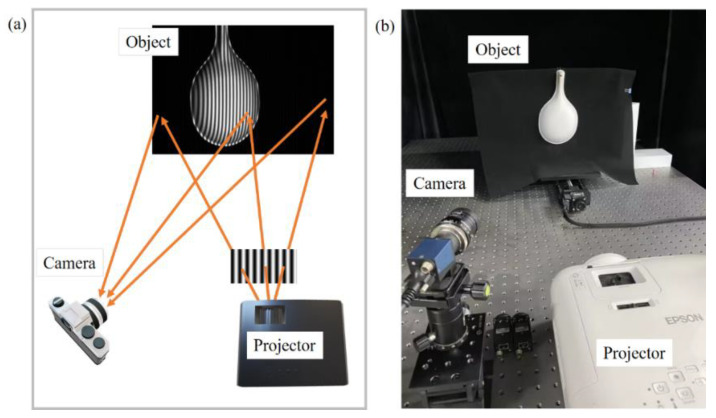
Experimental measurement system. (**a**) Schematic diagram. (**b**) Actual setup diagram.

To further verify the proposed method’s effectiveness, a fringe projection system was built and experiments were conducted, as shown in Figure 7. The fringe projection system consisted of a projector (Epson CH-TW5600) with a resolution of 1920 × 1080 and a camera (IMAVISION MER-231-41GM-P) with a resolution of 1920 × 1200. The four-step phase-shifting algorithm was used in the above discussion to solve the phase, and the Gray-code was adopted to unwrap the phase [25]. To eliminate the influence of the nonlinear response of the system, a grayscale lookup table was made by calibration in advance [26] so that there was an approximately linear response between the projector and the camera. In addition, because the real fringe was different from the simulated fringe, there was also the influence of nonlinear effects. Therefore, after obtaining the restored fringe, one could use Hilbert transform [27,28] to process all the fringe, then calculate the phase of the two separately and then average them, which effectively offsets the impact of nonlinearity.

**Figure 8:**
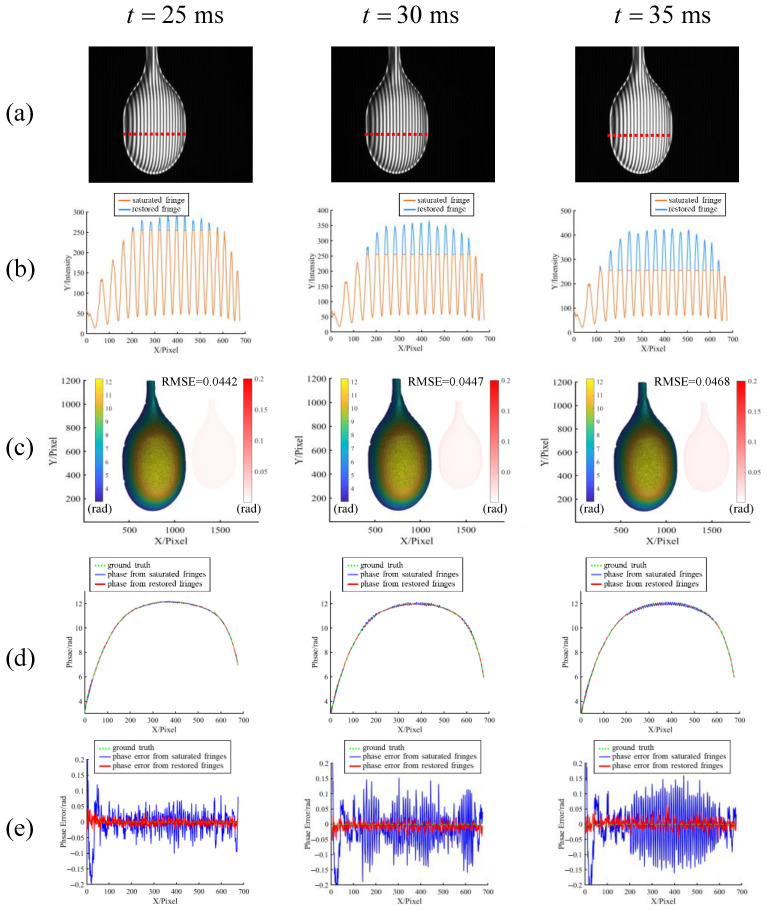
Experimental verification of the proposed method in the cases of saturation generated under different exposure times. The exposure times of the first, second, and third rows are 25 ms, 30 ms, and 35 ms, respectively. (**a**) Saturated fringes. (**b**) Comparison of the section lines of saturated fringe and restored fringe along the red dotted line in (**a**). (**c**) Phases calculated from the restored fringes; the small figures at the right sides represent the error maps. (**d**) Comparison of the section lines of phases calculated from saturated fringes, restored fringes, and ground truth along the red dotted line in (**a**). (**e**) Error comparison of (**d**).

The experiment started with a single ladle selected for verification. Different fringe saturation states were generated by changing the camera’s exposure time. The exposure time was set to 25 ms, 30 ms, and 35 ms, respectively, and the measured results are shown in Figure 8. Figure 8a shows the saturated fringe patterns obtained by the camera, and Figure 8b shows the section lines under different saturation states and their restored results. It can also be seen that more than 255 parts of the saturated fringe are truncated, and then the saturated part can be restored by the proposed method. Figure 8c shows the phase recovered using the restored fringes, and the small figures on the right are the difference from the ground truth, obtained under the unsaturated condition using a 24-step phase shift. It can be observed that with the increase of saturation degree, the restored phase is almost the same, and the difference to the ground truth is very small, proving the method’s robustness under different saturation degrees. Figure 8d shows the comparison of the section lines of phases calculated from saturated fringes, restored fringes, and ground truth, which are all sampled along the red dotted line in Figure 8a. Figure 8e shows the difference between the phases calculated and the ground truth in Figure 8d. It can be seen that as the degree of saturation increases, the fluctuation of the phase directly from the saturated fringe also gradually increases. Although the phase fluctuation of the proposed method is also increasing, it always remains at a low level and is far less than the former.

**Figure 9:**
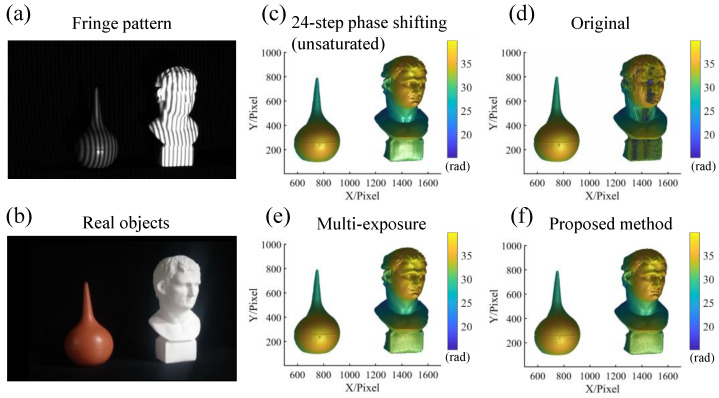
Comparison of experimental results of different methods. (**a**) Saturated fringes. (**b**) Real objects. (**c**) Ground truth was obtained by the 24-step phase-shifting method under unsaturated conditions. (**d**) Phase calculated directly from saturated fringes. (**e**) Phase recovered by the multiexposure method. (**f**) Phase recovered by the proposed method.

**Figure 10:**
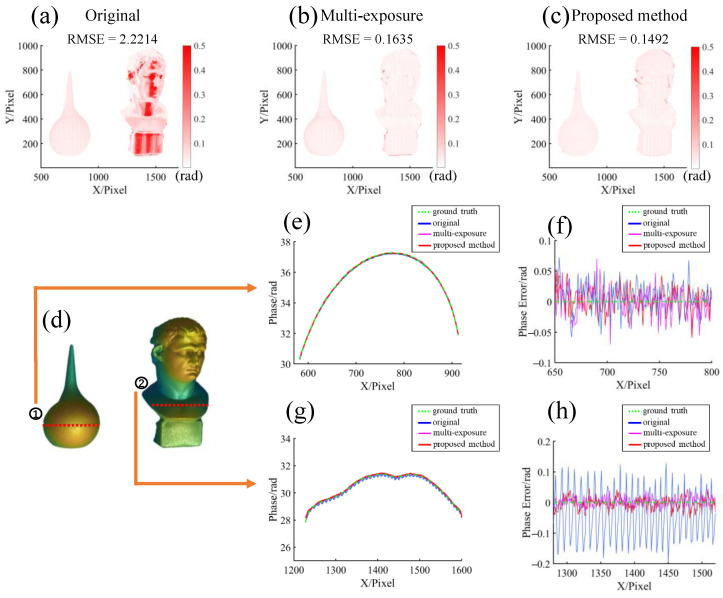
Error comparison of different methods. (**a**) Phase error directly from saturated fringes. (**b**) Phase error of multiexposure method. (**c**) Phase error of the proposed method. (**d**) Sampling positions of section lines. Panels (**e**,**f**) are the phase and error comparisons of different methods at sampling position 1, respectively. Panels (**g**,**h**) are the phase and error comparisons of different methods at sampling position 2, respectively.

To further verify the applicability of the proposed method, a high dynamic scene was selected for measurement, including an ear wash ball and a statue. Because of its plastic material, the surface reflectivity of the ear wash ball was low. To measure the surface profiles of these two objects simultaneously, one strategy is to use longer exposure time or stronger illumination to make the fringes on the ear wash ball normal. Still, at this time, the fringes on the statue will fall into saturation. Therefore, the proposed method can be used for experimental verification, and the results can also be compared with those of other methods, as shown in Figure 9. Figure 9a shows one of the fringe patterns taken in a high dynamic scene. It can be seen that the fringes on the statue are saturated. It is worth mentioning that there is also a saturated bright spot on the top of the ear wash ball due to a specular reflection on its surface, causing the projector’s light to reflect into the camera directly. Figure 9b shows a color image of the measured object, which clearly shows the characteristics of these two objects. The ear wash ball is a reddish-brown rubber with a smooth surface and weak specular reflection, while the statue is a white resin with high reflectivity and belongs to a diffuse reflection object. Figure 9c shows the phase obtained using a 24-step phase shift in the unsaturated condition, which is regarded as the ground truth and is used for the calculation of the error map and RMSE in Figure 10. Figure 9d–f shows the phase calculated directly using the original saturated fringes, the phase obtained by the multiexposure method [9], and the phase obtained by the proposed method, respectively. In Figure 9d, because the exposure on the ear wash ball is basically normal, the result is similar to the ground truth. However, the exposure on the statue is saturated, so the phase fluctuation is severe. The result in Figure 9e is from the multiexposure method, and its phase is very close to the ground truth. Still, it should be noted that the multiexposure method uses five groups of four-step phase-shifting fringes in the experiment. The proposed method also obtained results close to the ground truth, but only four conventional fringes were used; thus, the projection number was significantly lower than the 24 used for the ground truth and 20 used for the multiexposure method. In addition, it can also be found through comparison that the proposed method can obtain better results than those of other methods for the saturated area at the top of the ear wash ball, which shows the good applicability of this method for saturation treatment.

A more detailed quantitative comparison of the above different methods is shown in Figure 10. Figure 10a–c shows the error maps of each method compared with the ground truth, which can be seen to be consistent with the subjective conclusions drawn in Figure 9. The results of the original saturated fringe have very serious errors in the saturated area, while the results of the multiexposure method and the proposed method have smaller errors, and more significant errors appear at the edge. The RMSE of the proposed method is slightly smaller than that of the multiexposure method. Sampling lines are selected on the two objects to compare the results in more detail, as shown in Figure 10d. Figure 10e,f show the results of different methods and their errors on the sampling line of the ear wash ball, while Figure 10g,h show those of the statue. Because the exposure on the ear wash ball is within the normal range, the error level of the results of several methods is basically the same. Due to the large area of saturation on the statue, the phase calculated using the original fringe shows a very sharp fluctuation. It is worth noting that this periodic fluctuation is caused by the truncation of the saturated fringe itself, rather than the order jump caused by the phase unwrapping error. The multiexposure method and the proposed method significantly reduce that, showing almost the same error level.

## 5. Conclusions

This study proposed a saturated fringe restoration method for the possible saturation in FPP in high dynamic scenes. This method’s key is solving the parameter *A* related to the object’s reflectivity. According to the saturation of the fringe group, three area divisions are proposed: reliable area, shallow saturated area, and deep saturated area. The *A* in the corresponding area can be obtained by direct solution, CSI, and BSI, respectively. After *A* is restored, the proposed method can be used for fringe restoration. If there are still unrecovered parts in the fringe, we can further use CSI to complete, then restore the same part of the symmetrical fringe. For the experimental scene, the Hilbert transform can be applied to the restored fringes to reduce the impact of nonlinear error further. Through simulation and experimental verification, and compared with other methods, the proposed method can still obtain correct results without adding additional equipment or increasing projection number, so it has the potential to be applied to high-speed measurement occasions. Furthermore, although this paper is an example of a four-step phase shift, it can also be extended to a three-step phase shift and other steps similarly.

## Figures and Tables

**Table 1:** All saturation cases in four-step PSP.

Cases	Description	Specific Expression	Appearance Possibility	Solve *A*	Area Division
1	No saturation	$I_{1},I_{2},I_{3},I_{4}<255$	√	$(I_{1}+I_{2}+I_{3}+I_{4})/4$	Reliable area $\left(A<255\right)$
2	One saturated and three unsaturated	$I_{2},I_{3},I_{4}<255\;\&\;I_{1}\geq 255$	√	$(I_{2}+I_{4})/2$	
		$I_{1},I_{3},I_{4}<255\;\&\;I_{2}\geq 255$	√	$(I_{1}+I_{3})/2$	
		$I_{1},I_{2},I_{4}<255\;\&\;I_{3}\geq 255$	√	$(I_{2}+I_{4})/2$	
		$I_{1},I_{2},I_{3}<255\;\&\;I_{4}\geq 255$	√	$(I_{1}+I_{3})/2$	
3	Two saturated and two unsaturated	$I_{1},I_{2}<255\;\&\;I_{3},I_{4}\geq 255$	√	Unable	Shallow saturated area $\left(A^{\prime }<255\right)$ * or Deep saturated area $\left(A^{\prime }\geq 255\right)$ *
		$I_{1},I_{3}<255\;\&\;I_{2},I_{4}\geq 255$	×	/	
		$I_{1},I_{4}<255\;\&\;I_{2},I_{3}\geq 255$	√	Unable	
		$I_{2},I_{3}<255\;\&\;I_{1},I_{4}\geq 255$	√	Unable	
		$I_{2},I_{4}<255\;\&\;I_{1},I_{3}\geq 255$	×	/	
		$I_{3},I_{4}<255\;\&\;I_{1},I_{2}\geq 255$	√	Unable	
4	Three saturated and one unsaturated	$I_{1}<255\;\&\;I_{2},I_{3},I_{4}\geq 255$	√	Unable	
		$I_{2}<255\;\&\;I_{1},I_{3},I_{4}\geq 255$	√	Unable	
		$I_{3}<255\;\&\;I_{1},I_{2},I_{4}\geq 255$	√	Unable	
		$I_{4}<255\;\&\;I_{1},I_{2},I_{3}\geq 255$	√	Unable	
5	All saturated	$I_{1},I_{2},I_{3},I_{4}\geq 255$	√	Unable	

* Assume that *A*′ is the actual *A*, although it cannot be solved by formulas in cases 3 to 5.

## Data Availability

Not applicable.
