# Correction: Li et al. Fringe Projection Profilometry Based on Saturated Fringe Restoration in High Dynamic Range Scenes. *Sensors* 2023, *23*, 3133

**DOI:** 10.3390/s23135927

**Published:** 2023-06-26

**Authors:** Hongru Li, Hao Wei, Jiangtao Liu, Guoliang Deng, Shouhuan Zhou, Wenwu Wang, Liang He, Peng Tian

**Affiliations:** 1 College of Electronics and Information Engineering, Sichuan University, Chengdu 610065, China; lihoru99@scu.edu.cn (H.L.); shuairan@stu.scu.edu.cn (H.W.); liujiangtao@stu.scu.edu.cn (J.L.); gdeng@scu.edu.cn (G.D.); zhoush@scu.edu.cn (S.Z.); 2 School of Mechanical Engineering, Sichuan University, Chengdu 610065, China; wangwenwu@stu.scu.edu.cn (W.W.); hel20@scu.edu.cn (L.H.)

The authors wish to make the following corrections to the original paper [1].

## 1. Abstract Correction

There is a minor mistake in the 8th row of the abstract, the abbreviation “CSI” should be moved after “cubic spline interpolation”, The corrected text is shown below.

**Abstract**: In high dynamic scenes, fringe projection profilometry (FPP) may encounter fringe saturation, and the phase calculated will also be affected to produce errors. This paper proposes a saturated fringe restoration method to solve this problem, taking the four-step phase shift as an example. Firstly, according to the saturation of the fringe group, the concepts of reliable area, shallow saturated area, and deep saturated area are proposed. Then, the parameter *A* related to the reflectivity of the object in the reliable area is calculated to interpolate *A* in the shallow and deep saturated areas. The theoretically shallow and deep saturated areas are not known in actual experiments. However, morphological operations can be used to dilate and erode reliable areas to produce cubic spline interpolation (CSI) areas and biharmonic spline interpolation (BSI) areas, which roughly correspond to shallow and deep saturated areas. After *A* is restored, it can be used as a known quantity to restore the saturated fringe using the unsaturated fringe in the same position, the remaining unrecoverable part of the fringe can be completed using CSI, and then the same part of the symmetrical fringe can be further restored. To further reduce the influence of nonlinear error, the Hilbert transform is also used in the phase calculation process of the actual experiment. The simulation and experimental results validate that the proposed method can still obtain correct results without adding additional equipment or increasing projection number, which proves the feasibility and robustness of the method.

## 2. Figure Correction

There was a mistake in Figure 6 due to information going missing in the publication stage. The corrected Figure 6 is shown below.

## 3. Funding Correction

The following funding information was not included in the original paper, which has been added: Postdoctoral Research and Development Fund of Sichuan University (2023SCU12008). The corrected funding is shown below.

**Funding:** Sichuan Province Science and Technology Support Program (2021YFG0323); Postdoctoral Research and Development Fund of Sichuan University (2023SCU12008); Postdoctoral interdisciplinary innovation initiation fund of Sichuan University (2019BHJC11).

The authors apologize for any inconvenience caused and state that the scientific conclusions are unaffected. This correction was approved by the Academic Editor. The original publication has also been updated.

## Figures and Tables

**Figure 6:**
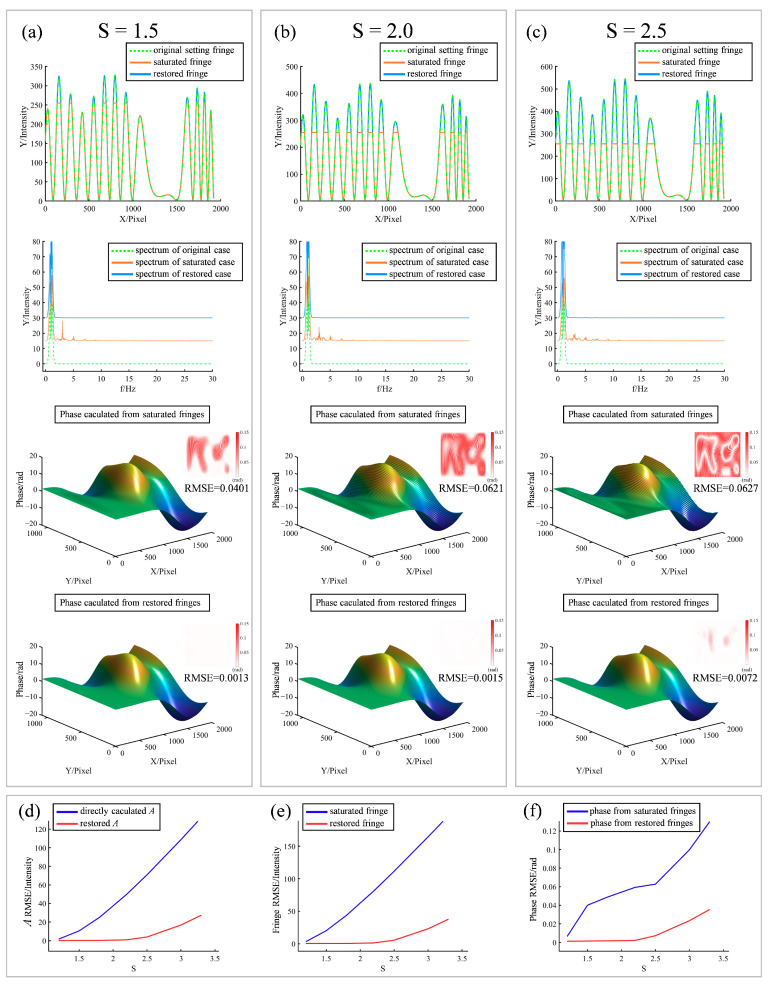
Simulation verification of the proposed method. Panels (**a**–**c**) are the cases when S = 1.5, 2.0, 2.5 , respectively. The first row is the comparison of the section lines of the original setting fringe, saturated fringe, and restored fringe. The second row is the comparison of the spectra of the original, saturated, and restored cases. The third row is the phase calculated directly from the saturated fringes, and the fourth row is the phase calculated using the restored fringes. The small figures in the upper right corners of the third and fourth rows indicate the error maps. (**d**) Error comparison between the directly calculated A and restored A that changed with S . (**e**) Error comparison between the saturated fringe and restored fringe that changed with S . (**f**) Error comparison between the phases obtained directly from the saturated fringes and those from restored fringes that changed with S .
